# Post-procedural Thyroid Cyst Infection Following Aspiration and Polidocanol Sclerotherapy: A Case Report

**DOI:** 10.7759/cureus.105917

**Published:** 2026-03-26

**Authors:** Sigfrido Miracle López, Marco-Antonio Alvarez-Arrazola

**Affiliations:** 1 Endocrinology, Hospital Angeles de las Lomas, Huixquilican, MEX; 2 Center for Health Sciences Research, Universidad Anahuac Mexico Norte, Huixquilucan, MEX; 3 Radiology, Universidad Politecnica de Sinaloa, Mazatlan, MEX

**Keywords:** acute suppurative thyroiditis, case report, lauromacrogol 400, polidocanol, sclerotherapy, thyroid abscess, thyroid cyst, thyroid nodule, ultrasound-guided aspiration

## Abstract

Ultrasound-guided aspiration and sclerotherapy are established minimally invasive approaches for the management of benign cystic and predominantly cystic thyroid nodules. Polidocanol has been reported as an effective sclerosant with a favorable safety profile, although infectious complications are rarely emphasized. We describe a 65-year-old woman with type 2 diabetes mellitus and rheumatoid arthritis who underwent ultrasound-guided aspiration of a recurrent predominantly cystic left thyroid nodule, with drainage of 33 cc of thick colloid material, intracavitary instillation of 4 cc of 3% polidocanol, and fine-needle aspiration of the residual solid component. Within 24 hours, she developed fever and systemic symptoms, initially without obvious local inflammatory signs. Over the following days, she developed painful left cervical swelling and marked sonographic re-expansion of the lesion. Repeat aspiration yielded purulent-appearing material, although only 5 cc could be drained despite multiple attempts. Laboratory testing showed a leukocyte count of 12,100/µL, total neutrophils of 9,860/µL, total lymphocytes of 1,940/µL, immature granulocytes of 0, an erythrocyte sedimentation rate of 12 mm/h, and a C-reactive protein level of 19.230 mg/dL. Gram stain demonstrated gram-positive cocci, culture grew *Staphylococcus epidermidis*, and anaerobic culture at seven days was negative. The patient subsequently required hospitalization for 72 hours and intravenous trimethoprim-sulfamethoxazole, with clinical improvement and reduction in cervical swelling. This case highlights a rare probable post-procedural infectious complication after aspiration and polidocanol sclerotherapy of a predominantly cystic thyroid nodule and underscores the value of early repeat ultrasound and microbiologic reassessment when persistent fever, pain, and rapid lesion re-expansion occur.

## Introduction

Thyroid nodules are common in clinical practice and population-based imaging studies. Their prevalence varies according to the detection method, ranging from approximately 2% to 6% by palpation and substantially higher rates by ultrasonography and autopsy [1]. Cystic and predominantly cystic nodules constitute an important subgroup because they are usually benign but may become clinically relevant due to compressive symptoms, local discomfort, or cosmetic concerns [1,2]. A predominantly cystic thyroid nodule is generally understood as a nodule in which the cystic component accounts for most of the lesion volume, typically more than 50%.

Simple aspiration can provide temporary decompression, but recurrence is common [2,3]. For recurrent benign cystic or predominantly cystic thyroid nodules, image-guided ablation techniques have gained an increasingly important role. In this context, sclerotherapy refers to ultrasound-guided evacuation of cyst contents followed by instillation of a sclerosant to promote fibrosis of the cavity and reduce recurrence. Ethanol ablation remains the best-established nonsurgical option, but polidocanol (lauromacrogol 400), a detergent sclerosant, has also shown favorable results in selected series, with meaningful volume reduction and a low rate of adverse events [2-6]. Current European Thyroid Association guidance recognizes image-guided ablation as an accepted treatment strategy for selected benign thyroid nodules [6].

Thyroid infection is rare because the gland is relatively resistant to bacterial invasion owing to its rich vascular and lymphatic supply, high iodine content, and fibrous capsule [7-9]. Nevertheless, acute suppurative thyroiditis and thyroid abscess remain clinically important because delayed recognition may lead to significant morbidity [7-9]. Prior instrumentation, including fine-needle aspiration, has been described as a rare predisposing factor for thyroid infection [7-10]. Published reports of infectious complications after percutaneous treatment of cystic thyroid lesions are limited to isolated cases and rare events within larger series [10-12]. We report a post-procedural thyroid cyst infection following aspiration and polidocanol sclerotherapy of a predominantly cystic thyroid nodule, with emphasis on the imaging evolution and the practical distinction between true infection and possible specimen contamination.

## Case presentation

A 65-year-old woman with a history of type 2 diabetes mellitus and rheumatoid arthritis was evaluated for a recurrent predominantly cystic left thyroid nodule. At the initial consultation, she reported having noticed progressive anterior neck enlargement approximately eight months before the procedure. According to the written report of a thyroid ultrasound performed at an outside center in May 2025, the right thyroid lobe contained three round anechoic lesions with posterior acoustic enhancement measuring 1, 2, and 4 mm, consistent with simple cysts. The left thyroid lobe was reported to contain two oval-shaped, well-defined, anechoic lesions with irregular borders measuring 25 × 18 mm and 30 × 26 mm. The original ultrasound images from that outside study were not available for review or inclusion in this manuscript. The patient also stated that a fine-needle aspiration had been performed on September 12, 2025, with cytology reported as Bethesda II, consistent with lymphocytic thyroiditis and follicular nodular disease.

Her type 2 diabetes mellitus had been diagnosed in 2018 and was being treated with metformin 425 mg daily and linagliptin 5 mg daily at the time of the procedure. Laboratory testing performed three months before the intervention showed a fasting glucose level of 118 mg/dL (reference range, 70-99 mg/dL) and an HbA1c of 6.1% (reference range, 4.0%-5.6%). She also had rheumatoid arthritis diagnosed in 2025, treated with hydroxychloroquine once daily; the exact dose was not available in the reviewed records. At the time of the procedure, the patient denied receiving corticosteroids or any additional disease-modifying antirheumatic drug therapy.

**Figure 1:**
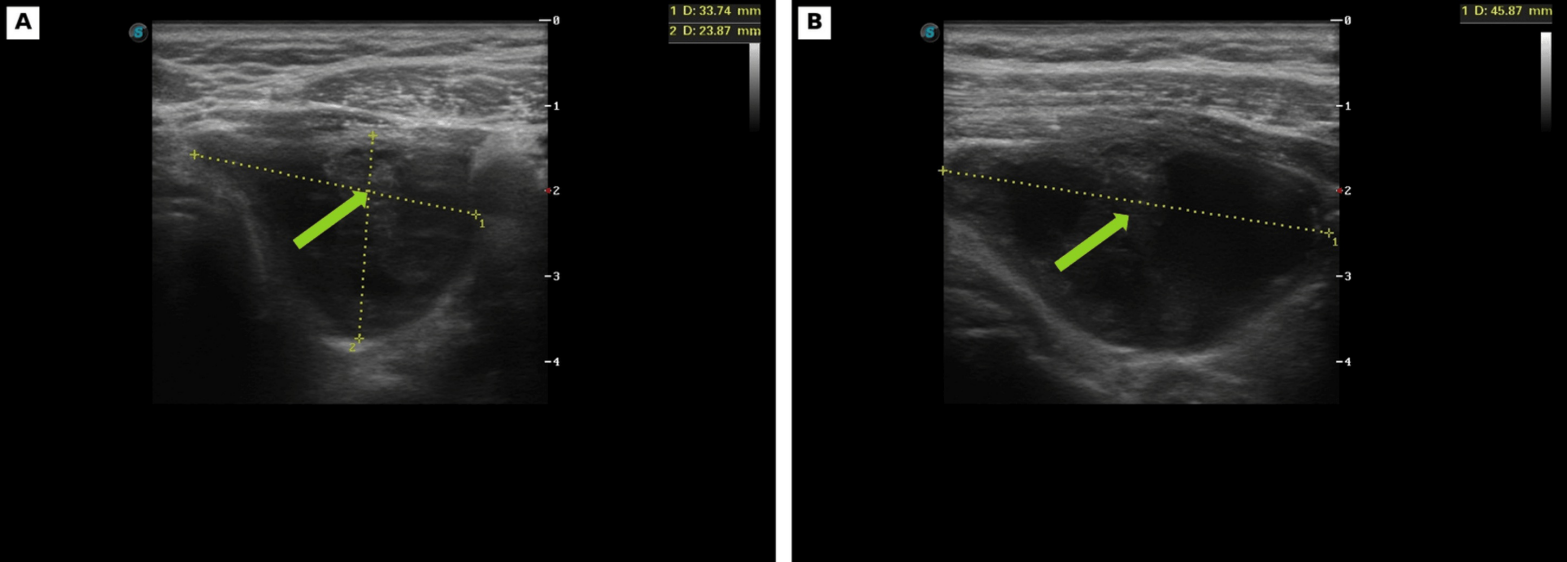
Pre-procedural ultrasound of the left thyroid lobe. (A-B) Representative grayscale images demonstrate a recurrent predominantly cystic lesion with internal echogenic debris and a residual mural or solid component. Arrows indicate the residual mural/solid component and internal echogenic material within the lesion.

Pre-procedural ultrasound performed at our center demonstrated a recurrent predominantly cystic lesion in the left thyroid lobe with internal echogenic debris and a residual mural or solid component, measuring approximately 45.9 × 33.7 × 23.9 mm in representative views (Figure 1A-1B). Given the lesion’s recurrent nature and symptomatic cervical enlargement, ultrasound-guided aspiration with sclerotherapy was performed in a single outpatient session.

**Figure 2:**
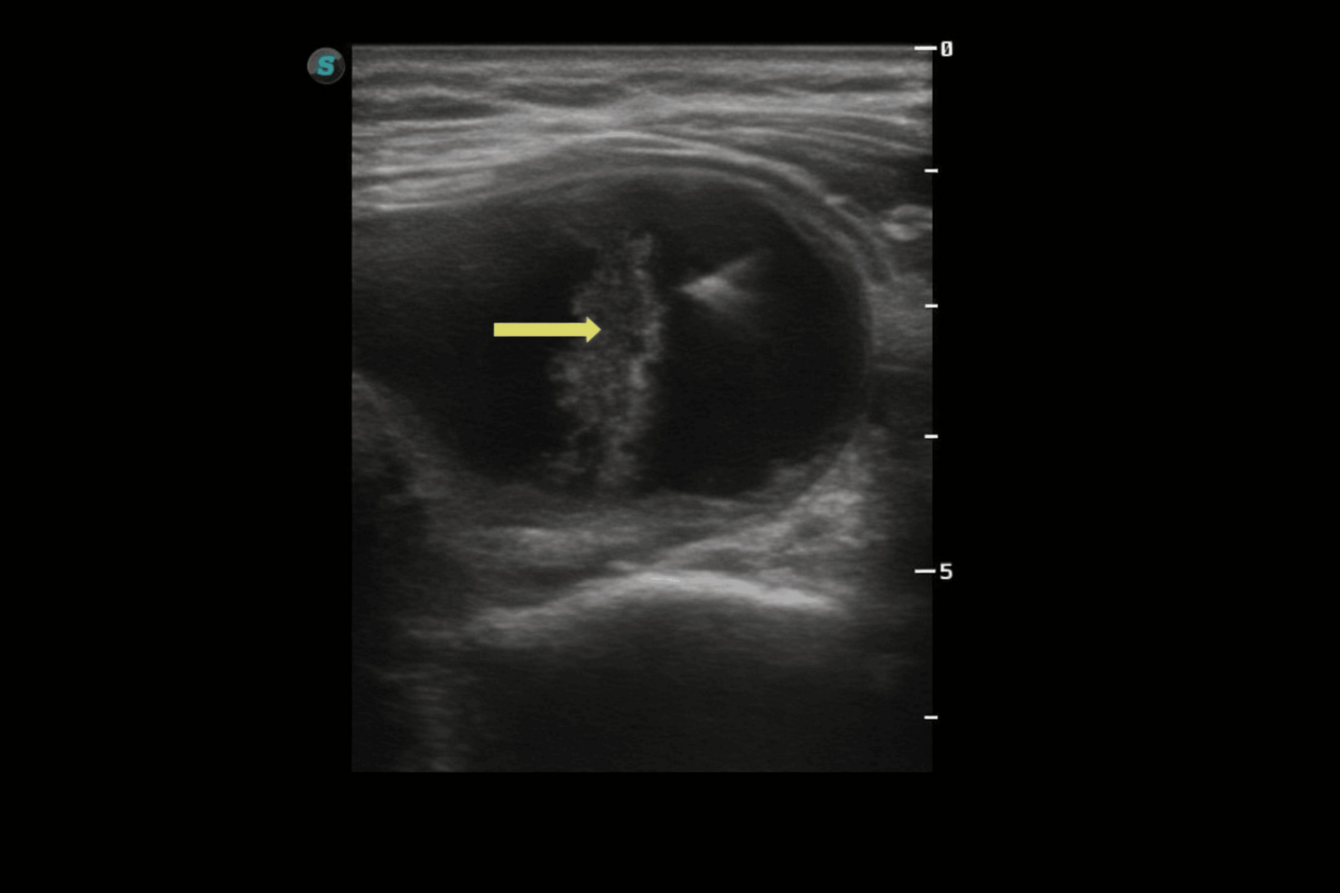
Intra-procedural ultrasound image. Representative image obtained during ultrasound-guided aspiration and fine-needle sampling, confirming the mixed cystic-solid morphology of the lesion. The arrow indicates the residual solid component targeted during sampling.

**Figure 3:**
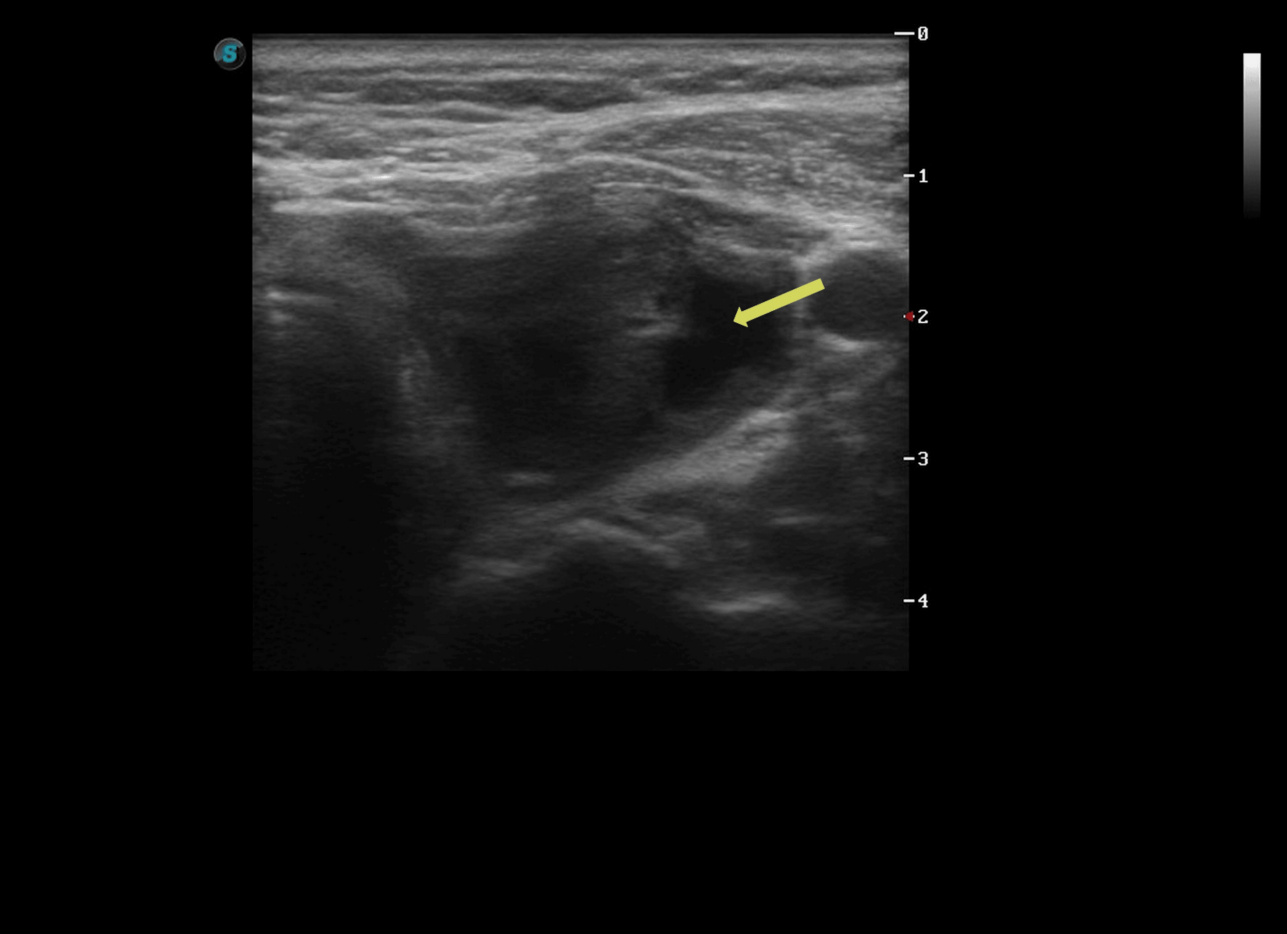
Immediate post-procedural ultrasound. Representative grayscale image showing partial collapse of the cystic cavity with residual echogenic material and no obvious extrathyroidal fluid collection on the available view. The arrow indicates the partially collapsed residual cavity with echogenic material.

The anterior neck region was prepared using Microdacyn® antiseptic solution (Sanfer Laboratories, Mexico City, Mexico), a hypotonic superoxidized solution. Topical analgesia with lidocaine/prilocaine cream (EMLA®, Aspen Pharma, Durban, South Africa; 25 mg/25 mg) was applied to the skin before puncture, and the thyroid capsule was infiltrated with 2% lidocaine. A 25-gauge needle was used for cyst aspiration, which was performed in two passes. A total of 33 cc of thick colloid material was aspirated. Subsequently, 4 cc of 3% polidocanol (lauromacrogol 400; Aethoxysklerol, Kreussler Pharma, Wiesbaden, Germany) was instilled into the cavity and left in situ. Fine-needle aspiration of the solid component was also performed using a 25-gauge needle. Multiple needle trajectories were used during the procedure, including two for drainage of the cystic component and one for aspiration of the solid component. Intra-procedural ultrasound confirmed the mixed cystic-solid appearance of the lesion (Figure 2). Immediate post-procedural ultrasound showed partial collapse of the cystic cavity, residual echogenic material, and no obvious extrathyroidal fluid collection on the available views (Figure 3). No technical difficulty or immediate bleeding was observed.

On post-procedure day 1, the patient contacted the treating team because of malaise, back pain, headache, left otalgia, and fever to 38.1°C. She denied pain, erythema, warmth, or swelling at the puncture site. Oral amoxicillin-clavulanate and acetaminophen were started, laboratory tests were requested, and warning signs were reviewed. Notably, the earliest post-procedural manifestations were predominantly systemic and nonspecific, without overt local inflammatory signs.

**Figure 4:**
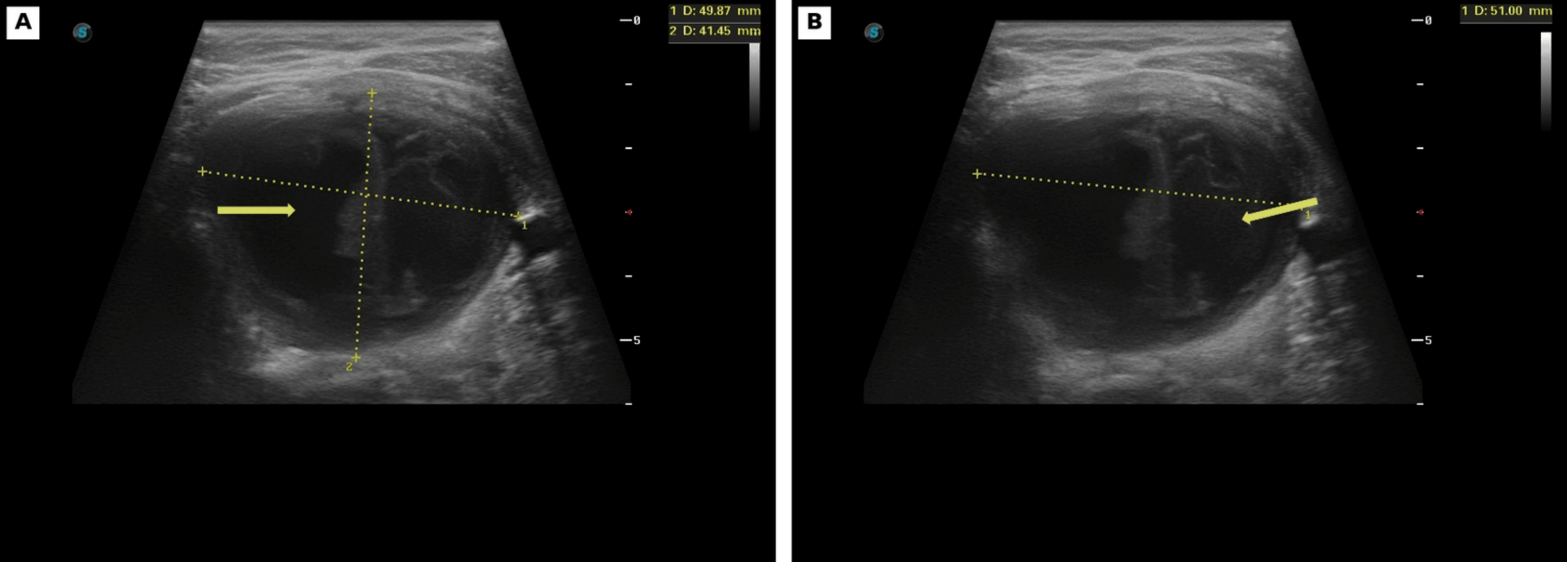
Follow-up ultrasound five days after the procedure. (A-B) Representative images show marked re-expansion of the predominantly cystic lesion compared with the immediate post-procedural study. Arrows indicate the re-expanded cystic lesion.

Approximately 72 hours later, she reported partial symptomatic improvement on antibiotics but persistent fever to 38.0°C, together with new cervical swelling and subjective re-expansion of the lesion. On repeat clinical assessment, she had persistent fever and tenderness over the left thyroid lobe. Follow-up ultrasound performed five days after the procedure demonstrated marked re-expansion of the predominantly cystic lesion, measuring approximately 49.9 × 41.5 × 51.0 mm (Figure 4A-4B).

**Figure 5:**
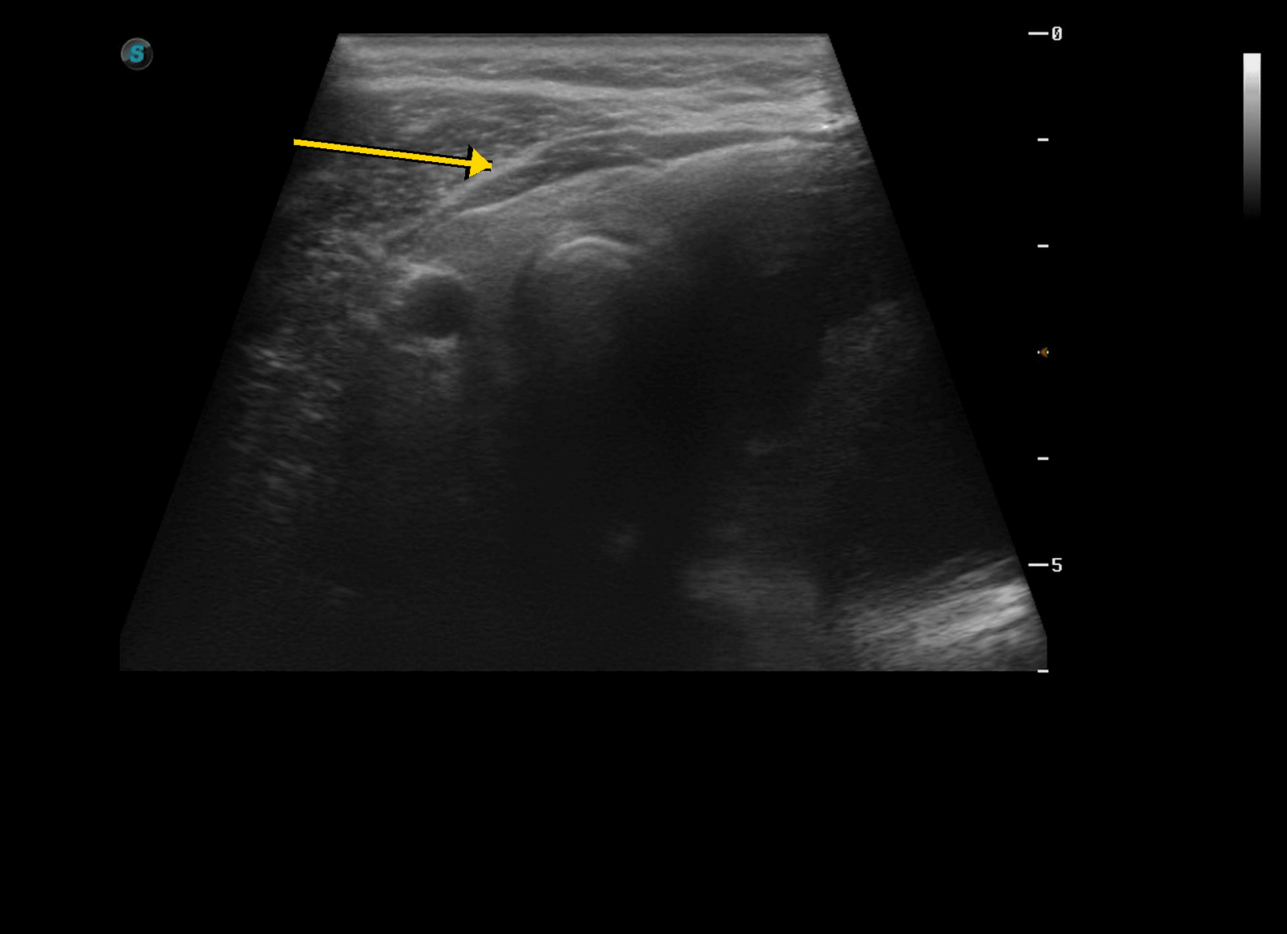
Follow-up ultrasound focused on the surrounding neck soft tissues. Representative image showing no clearly defined extrathyroidal fluid collection on the available sonographic views. The arrow indicates the perithyroidal soft-tissue region evaluated for possible extrathyroidal collection, suggesting that the process remained predominantly intrathyroidal on the evaluated images.

**Figure 6:**
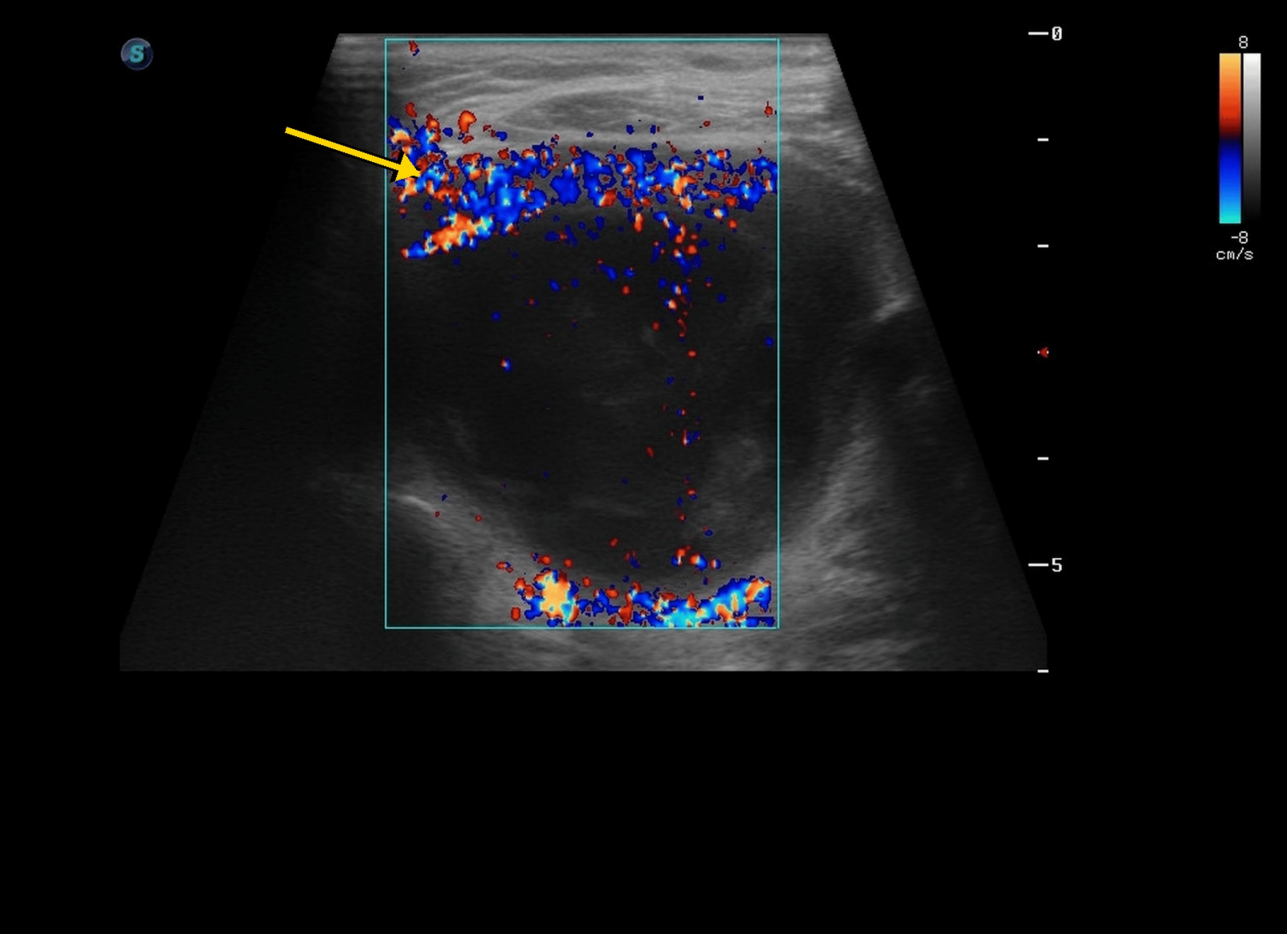
Color Doppler ultrasound of the re-expanded lesion. Representative Doppler image demonstrating predominantly peripheral vascularity along the lesion margin without marked internal hypervascularity. The arrow indicates the predominantly peripheral vascularity. This pattern is nonspecific and does not exclude infection, but it may be compatible with a re-expanded or partially organized cystic collection rather than a solid hypervascular inflammatory mass.

No clearly defined extrathyroidal fluid collection was evident on the available sonographic views (Figure 5). Color Doppler showed predominantly peripheral vascularity without striking internal hypervascularity (Figure 6). These later findings indicated progression from a nonspecific early post-procedural syndrome to a more localized thyroid process.

Because of the combination of persistent fever, local pain, inflammatory laboratory abnormalities, and rapid cyst re-expansion, repeat ultrasound-guided aspiration was performed. Purulent-appearing material was obtained and submitted for cytology, Gram stain, and culture; however, despite multiple attempts during this second procedure, only 5 cc could be drained. Cytologic examination of the repeat aspirate was reported as Bethesda I and demonstrated proteinaceous material with erythrocytes, scattered lymphocytes, and foamy macrophages, some showing phagocytic activity. Follicular cells were intentionally sought but were not identified. Overall, the specimen was interpreted as compatible with benign cyst contents. Gram stain demonstrated gram-positive cocci, and culture grew *Staphylococcus epidermidis *(*S. epidermidis*). An anaerobic culture obtained at seven days was negative. Laboratory testing at that time showed a leukocyte count of 12,100/µL, total lymphocytes of 1,940/µL, total neutrophils of 9,860/µL, immature granulocytes of 0, erythrocyte sedimentation rate of 12 mm/h, and C-reactive protein of 19.230 mg/dL, supporting an inflammatory/infectious process.

Given the clinical progression, imaging findings, and microbiologic results, the patient was referred for Infectious Diseases evaluation and was subsequently hospitalized at an outside institution for intravenous antibiotic treatment. She remained hospitalized for 72 hours and was treated with intravenous trimethoprim-sulfamethoxazole. During hospitalization, she experienced improvement in symptoms and a reduction in cervical swelling. However, no follow-up ultrasound had been performed at the time of manuscript preparation. A chronological summary of the patient’s clinical course is provided in Table 1.

**Table 1:** Timeline of the patient’s clinical course. FNA: fine-needle aspiration; HbA1c: glycated hemoglobin

Time point	Clinical event	Key findings	Intervention/outcome
May 2025	Thyroid ultrasound at an outside center	Right lobe: three simple cysts measuring 1, 2, and 4 mm. Left lobe: two oval-shaped anechoic lesions with irregular borders measuring 25 × 18 mm and 30 × 26 mm	Baseline imaging documented multinodular cystic thyroid disease
September 12, 2025	Fine-needle aspiration at an outside center	Cytology: Bethesda II, lymphocytic thyroiditis and follicular nodular disease	Conservative follow-up
Approximately June 2025 to February 2026	Progressive anterior neck enlargement	Patient reported gradual neck swelling over approximately eight months	Referred for further evaluation
Three months before procedure	Baseline metabolic assessment	Fasting glucose 118 mg/dL; HbA1c 6.1%	Diabetes considered reasonably controlled on oral therapy
February 2026 (day 0)	Evaluation at our center and ultrasound-guided aspiration with polidocanol sclerotherapy	Recurrent predominantly cystic left thyroid lesion measuring approximately 45.9 × 33.7 × 23.9 mm	33 cc thick colloid aspirated; 4 cc of 3% polidocanol instilled and left in situ; FNA of solid component performed
Immediate post-procedure	Post-procedural ultrasound	Partial collapse of the cystic cavity; no obvious extrathyroidal fluid collection	Patient discharged with routine precautions
Post-procedure day 1	Early symptoms reported	Malaise, back pain, headache, left otalgia, fever to 38.1°C; no local inflammatory signs	Oral amoxicillin-clavulanate and acetaminophen started
Approximately 72 hours after procedure	Persistent symptoms with new local findings	Persistent fever to 38.0°C, new cervical swelling, subjective lesion re-expansion	Repeat evaluation arranged
Post-procedure day 5	Follow-up ultrasound	Marked re-expansion of lesion to approximately 49.9 × 41.5 × 51.0 mm; no clear extrathyroidal collection; predominantly peripheral vascularity on Doppler	Repeat ultrasound-guided aspiration performed
Second aspiration	Sampling of re-expanded lesion	Purulent-appearing material obtained; despite multiple attempts, only 5 cc drained	Material sent for cytology, Gram stain, and culture
Same period	Cytologic and microbiologic results	Cytology: Bethesda I; Gram stain: gram-positive cocci; culture: Staphylococcus epidermidis; anaerobic culture at seven days negative	Findings interpreted in clinical context as supportive of post-procedural infection
Same period	Laboratory reassessment	Leukocyte count 12,100/µL; total lymphocytes 1,940/µL; total neutrophils 9,860/µL; immature granulocytes 0; erythrocyte sedimentation rate 12 mm/h; C-reactive protein 19.230 mg/dL	Supported inflammatory/infectious process
Subsequent hospitalization	Admission to outside institution	Persistent clinical progression	Hospitalized for 72 hours; treated with intravenous trimethoprim-sulfamethoxazole
After hospitalization	Early clinical outcome	Improvement in symptoms and reduction in cervical swelling	No follow-up ultrasound had been performed at time of manuscript preparation

## Discussion

Ultrasound-guided aspiration and sclerotherapy are established minimally invasive options for the management of benign cystic and predominantly cystic thyroid nodules, particularly in patients with local symptoms or cosmetic concerns. Simple aspiration alone is associated with a high recurrence rate, which has driven the use of sclerosant agents to improve durability. In published series, polidocanol has shown favorable volume reduction and a low rate of adverse events, with most reported complications being mild and self-limited, such as transient local pain, low-grade fever, swallowing discomfort, or minor bleeding. Major infectious complications have not been emphasized in the available polidocanol series, making the present case clinically notable [2-6].

Thyroid infection remains uncommon because the gland is relatively resistant to bacterial invasion owing to its rich blood supply, lymphatic drainage, iodine content, and encapsulation. Even so, acute suppurative thyroiditis and thyroid abscess can occur in the setting of pre-existing thyroid disease, congenital anomalies such as pyriform sinus fistula, or host-related risk factors including diabetes and immunocompromised states. Prior instrumentation, including thyroid fine-needle aspiration, is also a recognized but rare precipitating event. Reviews and case reports indicate that post-fine-needle aspiration (FNA) infection may present within a few days or, less commonly, weeks to months after the procedure [7-10].

The present case is unusual because the suspected infectious complication occurred after a combined therapeutic and diagnostic intervention involving cyst aspiration, intracavitary polidocanol instillation, and aspiration of the residual solid component in the same session. The temporal relationship was strong: systemic symptoms began on the first post-procedural day, followed by persistent fever, local pain, and rapid cyst re-expansion over the ensuing days. Repeat aspiration yielded purulent-appearing material, although only 5 cc could be drained despite multiple attempts, suggesting a dense or partially organized collection rather than a simple recurrent serous reaccumulation. Quantitative laboratory findings further supported a clinically significant inflammatory process, including leukocytes 12,100/µL, total neutrophils 9,860/µL, total lymphocytes 1,940/µL, immature granulocytes 0, erythrocyte sedimentation rate 12 mm/h, and C-reactive protein 19.230 mg/dL. Together with the subsequent clinical response to intravenous antibiotics, these findings supported a probable post-procedural infectious/inflammatory complication rather than an uncomplicated post-procedural reaction.

An important interpretive issue in this case is the microbiologic significance of *S. epidermidis*. Because *S. epidermidis* is a common skin commensal, its recovery from aspirated material may represent contamination rather than a true pathogen. That caution is especially relevant in thyroid procedures, where inoculation of low-virulence skin flora is theoretically possible but culture contamination is also plausible. However, the culture result was not interpreted in isolation. In this patient, its potential clinical relevance was supported by several converging features: symptom onset within 24 hours of instrumentation, persistent fever, localized thyroid tenderness, rapid sonographic re-expansion of the lesion, purulent-appearing aspirate, gram-positive cocci on Gram stain, inflammatory laboratory abnormalities, and improvement after inpatient intravenous antibiotic therapy. For that reason, we believe the most defensible interpretation is that this was a probable post-procedural infectious complication, while still acknowledging that microbiologic certainty was limited and contamination cannot be fully excluded.

The differential diagnosis included sterile post-procedural inflammation, intracystic hemorrhage, and chemical irritation related to sclerosant therapy. These alternatives are relevant because pain, transient fever, and sonographic complexity may occur after minimally invasive thyroid procedures. Nevertheless, several features argued against a purely sterile process in this case: the persistence of fever despite initial oral therapy, the rapid re-expansion of the lesion, the purulent gross appearance of the aspirate, the positive Gram stain, and the systemic inflammatory response. It is also plausible that tissue injury related to needle manipulation and intracavitary sclerosant exposure created a local environment more susceptible to secondary infection, although causality cannot be proven from a single case. This interpretation should therefore be presented as a reasoned inference rather than a definitive mechanistic conclusion.

Management of acute suppurative thyroiditis and thyroid abscess should be individualized according to clinical severity, airway risk, response to antimicrobial therapy, and the feasibility of drainage. Earlier literature favored surgery more routinely, but more recent reports support less invasive management in selected patients, including antibiotics combined with ultrasound-guided aspiration or drainage. Surgery is generally reserved for patients with airway compromise, uncontrolled sepsis, persistent or recurrent collection not amenable to aspiration, associated structural pathology requiring resection, or failure of conservative therapy. In the present case, the patient improved with repeat aspiration and intravenous trimethoprim-sulfamethoxazole during a 72-hour hospitalization, and no immediate surgery was required [7-12].

This case has several limitations. Complete records from the outside hospitalization were not available, and the final inpatient imaging workup could not be reviewed. Likewise, no follow-up ultrasound had been performed at the time of manuscript preparation, which limits assessment of residual cavity size, complete resolution, or recurrence. In addition, although the clinical picture strongly supported infection, the isolation of *S. epidermidis* does not entirely exclude contamination. These limitations should temper the strength of the causal claim.

Despite these limitations, the case offers a practical message. Persistent fever, localized thyroid pain, and rapid re-expansion of a treated cystic lesion after aspiration and sclerotherapy should prompt early repeat imaging and consideration of repeat aspiration with microbiologic analysis. Even when the cultured organism is of uncertain pathogenicity, the full clinical context may still support early escalation of care. This point is particularly relevant because published polidocanol series emphasize overall safety, which may lower suspicion for rare infectious complications in day-to-day practice.

## Conclusions

Polidocanol sclerotherapy remains an effective minimally invasive treatment for benign cystic and predominantly cystic thyroid nodules and is generally regarded as safe. This case suggests that post-procedural infection should be considered when persistent fever, localized pain, and rapid lesion re-expansion occur after treatment. Because this is a single case report and microbiological causality cannot be established with certainty, particularly in the setting of *Staphylococcus epidermidis* isolation, the findings should be interpreted cautiously. Nevertheless, early repeat ultrasound, repeat aspiration when feasible, and microbiologic assessment may help identify clinically important complications and guide escalation of care. Additional case reporting will be important to clarify the frequency, mechanisms, and microbiologic spectrum of infection after thyroid aspiration and sclerotherapy.
